# The Clinical Significance and Potential Role of C-Reactive Protein and Albumin in Antineutrophil Cytoplasmic Antibody Associated Vasculitis

**DOI:** 10.31138/mjr.280124.tcs

**Published:** 2024-12-31

**Authors:** Zohreh Jadali

**Affiliations:** Department of Immunology, School of Public Health, Tehran University of Medical Sciences, Tehran, Iran

**Keywords:** antineutrophil cytoplasmic antibody (ANCA)-associated vasculitis, C-reactive protein, albumin, inflammation

## To the Editor:

I read the article by Atas et al. about the association between C-reactive protein (CRP) to albumin ratio (CAR) with disease activity in patients suffering from antineutrophil cytoplasmic antibody (ANCA)-associated vasculitis (AAV).^1^ These findings are important because there is not enough data yet to make a firm conclusion regarding the value of CAR measurement in AAV. Previous research indicating that CAR is an independent predictor of mortality in patients with newly diagnosed AAV.^2^ This finding is in line with results from other studies that represent CAR has favourable prognostic value in many inflammatory diseases.^3^ Therefore, assessment of these inflammatory markers can provide useful information about disease status.

Nonetheless, some important questions remain, especially concerning the physiological and pathophysiological implications of CRP and albumin in AAV. Unfortunately, there is no clear answer to this question because each of them can exhibit distinct functional properties and opposing biological activities. These features can make it difficult to interpret their mechanism of action.

CRP is a prototype marker of the inflammatory process, with pleiotropic activity on human cells. It is an intriguing protein with two conformationally distinct isoforms. Pentameric CRP (pCRP) that are formed by the association of five subunits and monomeric CRP (mCRP), as an inflammatory derivative of circulating pCRP. Conformational change of the pCRP to mCRP contribute to shaping the inflammatory response and biological function of CRP.^4^

pCRP, which is primarily found in plasma exhibits anti-inflammatory properties. These activities are essential for host defence and are ascribed to mechanisms such as enhancement of IL-1 receptor antagonist expression in human peripheral blood mononuclear cells, increasing the secretion of anti-inflammatory cytokines (e.g., IL-10), and down-regulation of proinflammatory cytokines including IL-12 and IFN-γ. pCRP binding to ligands such as phosphocholine or phosphoethanolamine head groups exposed on the surface of activated or damaged cells, results in formation of mCRP. In contrast to pCRP, mCRP, which is preferentially expressed in tissues, exhibits potent pro-inflammatory actions, through various mechanisms including stimulation of thrombosis, activation of immune cells (e.g., endothelial cells, monocytes, platelets and neutrophils), neutrophil and monocyte adhesion to endothelial cells, increased formation of neutrophil-platelet and platelet-monocyte complexes, production of pro-inflammatory cytokines (e.g., IL-1β and IL-6), and augmentation of respiratory burst.^5^

According to the versatile and opposing bioactivities of the two CRP isoforms, it seems that the use of CRP as a marker of inflammation should be interpreted cautiously and there is a clear need to determine the effects of each CRP isoform during the inflammation. This is because on the one hand, many previous studies did not specify which CRP isoform was measured and because on the other hand pCRP is classically quantified rather than mCRP for clinical purposes. So, further studies with proper consideration of the distinct isoforms provide a better knowledge about their molecular mechanism of action.

Serum albumin is another inflammation-related molecule which was assessed by this study. The authors found that patients with AAV had significantly low serum albumin levels. This is in line with some previous researches, however, several in vivo and ex vivo studies, indicating that the synthesis rate of albumin in response to severe inflammation may increase or remain unchanged.^6^ It is important to note that these measures come from only a single time point, while the measurement of protein abundances in the study and diagnosis of human disease requires analysis of more than one time point. This issue should not be underestimated because albumin synthesis may differ at different stages of the inflammatory process. Some studies that show a biphasic response of albumin synthesis in rat experimental model of sepsis provide support for this concept.^7^

Despite controversies around the quantitative changes in the concentration of albumin there is consensus that inflammatory response can alter the serum albumin levels. Serum albumin levels can have a massive impact on AAV pathophysiology, but study of its molecular mechanism is difficult because of its pleotropic properties.

Albumin has an oncotic role that is essential for the stabilisation of intravascular volumes. It also possesses antioxidant and anti-inflammatory properties, protect endothelial integrity, can regulate immune responses and is important as a transport protein. One of the most important things to consider, before interpreting and displaying the research results about the albumin quantification is the concept of ‘effective albumin concentration’. This term is used to describe the structurally and functionally intact content of albumin because alterations in the chemical structure of albumin affects its physicochemical properties. Therefore, the concentration of serum albumin cannot entirely reflect the albumin biological function. For this reason, albumin could act as a “double-face” protein in modulating the inflammatory response. On the one hand, the native form of albumin has been involved in multiple anti-inflammatory processes such as enhanced production of bioactive anti-inflammatory lipids (such as lipoxins, resolvins, and protectins), inhibition the inflammatory mediators (e.g., TNF-α and C5a) and suppression the production of proinflammatory prostaglandins, free radicals and cytokines.^8^ On the other hand, some post translational modifications of human serum albumin due to proinflammatory microenvironment may compromise its functions. These modifications can alter its anti-inflammatory activities and impairs its antioxidant functions.^9^ Therefore, determination of effective albumin concentration is another important issue that needs to be addressed now.

Overall, inflammation in patients with AAV is an important contributing factor of disease pathophysiology. Therefore, measurement of inflammation-related indicators could provide important insights into the pathogenesis of disease and its management. These markers can be used to determine the presence of inflammation and quantification of its severity. Of these CRP, albumin and CAR are the most commonly used markers. But until recently, it is not known whether these factors are disease markers, disease makers or both. So, further studies are needed to elucidate the exact role of these molecules in AAV.
